# Cortical Organization of Language Pathways in Children with Non-Localized Cryptogenic Epilepsy

**DOI:** 10.3389/fnhum.2014.00808

**Published:** 2014-10-09

**Authors:** Richard Eugene Frye, Jacqueline Liederman

**Affiliations:** ^1^Department of Pediatrics, University of Arkansas for Medical Sciences, Little Rock, AR, USA; ^2^Arkansas Children’s Hospital Research Institute, Little Rock, AR, USA; ^3^Department of Psychological and Brain Sciences, Boston University, Boston, MA, USA

**Keywords:** epilepsy, reading, language, magnetoencephalography, dynamic statistical parameter

## Abstract

Children with a history of epilepsy are almost six times more likely than their unaffected siblings to be referred for speech or language therapy. However, the abnormalities in neural pathway that cause these delays are poorly understood. We recorded evoked fields using whole-head magnetoencephalography during real and non-word visual and auditory rhyme tasks in 15 children with non-localized cryptogenic epilepsy. Basic phonological and orthographic language skills were assessed using Woodcock–Johnson Test of Achievement subtests. Dynamic statistical parameter mapping was used with individual participant magnetic resonance images. Significant cortical activity was visualized on average and performance weighted maps. For the auditory rhyme tasks, bilateral primary and secondary auditory cortices, the superior temporal sulcus, and insular cortex were activated early with later increases in left hemisphere activity. Visual rhyme tasks evoked early bilateral primary and secondary occipital cortical and angular gyri activity followed by later activation of the planum temporale and supramarginal gyri and the left ventral occipitotemporal area. For the auditory rhyme tasks, performance weighted maps demonstrated that early right hemisphere activation was associated with poorer reading skills while later activity was associated with better reading skills; for the left hemisphere, greater early activation of the secondary auditory cortex, including the planum temporale, was related to better reading skills while relatively later activation of these areas was associated with poorer reading skills. For the visual rhyme tasks, greater activity in the bilateral ventral occipitotemporal and insular areas and angular and supramarginal gyri were associated with better performance. These data suggest that spatiotemporal cortical activation patterns are associated with variations in language performance in non-localized cryptogenic epilepsy.

## Introduction

Seizures are the most common neurological disturbance of childhood. At least 8% of children will have a seizure, with most seizures being rather benign and limited to either febrile periods or an isolated occurrence (Berg et al., 2013). A smaller percentage of children (approximately 2%) will go on to have epilepsy (defined as two or more unprovoked seizures). Although it was once believed that the cognitive effects of most childhood epilepsies were minimal except in refractory and early onset epilepsies, it is being increasingly understood that childhood epilepsy is associated with cognitive, behavioral, and psychiatric consequences (Baxendale et al., 2010; Benn et al., 2010; Monjauze et al., 2011; Jambaque et al., 2013; Loughman et al., 2014; Melbourne Chambers et al., 2014). Indeed, Benn et al. (2010) reported that children with a history of epilepsy (even those with normal anatomical findings) were almost six times more likely to have been referred for treatment of speech/language problems than their unaffected siblings. Even benign rolandic epilepsy, a partial epilepsy believed not to be associated with cognitive abnormalities, has been linked to attention and learning abnormalities (Danielsson and Petermann, 2009; Pal, 2011; Miziara et al., 2012; Vannest et al., 2013).

The reason for cognitive abnormalities in children with epilepsy is not clear. While frequent seizures could result in frequent disruptions in cognitive neural networks, many other factors could be associated with problems in cognitive development. For example, epilepsies not associated with frequent seizures are also associated with cognitive abnormalities, antiepileptic drug treatments have adverse cognitive effects, and cognitive delays have been found to be present at onset of the epilepsy (Jackson et al., 2013). Other evidence suggests that cognitive deficits are independent of antiepileptic drug treatment and resolve with remission of the epilepsy (Verrotti et al., 2011). Thus, it is possible that subclinical epileptiform disturbances found in many children with epilepsy could be disrupting the development of a variety of cognitive networks (Nicolai et al., 2012). However, the significance of these epileptiform disruptions are controversial and are believed by some to be associative rather than causative in relation to cognitive and behavioral abnormalities (Pal, 2011). The fact that many of the cognitive, behavioral, and psychiatric abnormalities associated with childhood epilepsy continue long-term, into adulthood, suggests that cognitive, behavioral, and psychiatric abnormalities are more likely caused by reorganization of the brain’s neural networks as a consequence of epilepsy, rather than an effect of an active seizure disorder *per se* (Baxendale et al., 2010; Monjauze et al., 2011).

Usually neuroimaging tools are used to assess the possibility of surgical resection for treatment of drug-resistant epilepsy. In this context, neuroimaging tools help localize epileptiform foci as well as identify eloquent cortex (Hertz-Pannier et al., 2013). Many neuroimaging tools have also been used to understand the changes in the cognitive networks of the brain in individuals with epilepsy. These include anatomic neuroimaging tools such as structural magnetic resonance imaging (MRI) (Overvliet et al., 2013) and diffusion tensor imaging (Besseling et al., 2013a) and functional neuroimaging tools such as functional MRI (Besseling et al., 2013b; Vannest et al., 2013), electroencephalography, and magnetoencephalography (MEG) (Paetau and Mohamed, 2013). Functional neuroimaging tools are particularly useful for examining cognitive changes in the activation of the brain’s neural networks. Neurophysiological tools such as electroencephalography and MEG allow the study of cognitive processes such as visual or auditory language as the decoding of language, especially speech, requires very rapid discrimination of successive sounds at a millisecond time scale (Frye et al., 2009b). Cognitive processes that operate on a rapid time scale include language, which is one of the key cognitive processes important for communication and learning. As such, language should be a special priority in the study of children with epilepsy.

In this study, we investigate the organization of the brain’s neural networks within children with cryptogenic epilepsy. To this end, we used whole-head MEG to study the timing and organization of two major routes of language neural processing networks (lexical and sublexical) through two sensory routes (visual and auditory). The dual-route model of reading posits that there are two major routes for reading, lexical and sublexical (Coltheart et al., 2001; Nickels et al., 2008). We select this model because language, especially reading, uses a wide range of cortical areas and is exquisitely sensitive to brain connectivity, network organization, and spatiotemporal dynamics of brain activation (Frye et al., 2009b), given that epilepsy can disrupt these aspects of brain development (Hamberger and Cole, 2011). Similar aspects of brain dynamics can also be disrupted in auditory language tasks that require phonological processing (Frye et al., 2008, 2009b).

To this end, we used real words and non-words in separate visual and auditory rhyming tasks. In the visual rhyming tasks, words were not spelled the same way, so phonological reading pathways needed to be engaged to decode the sound corresponding to the words. By using both real words and non-words, we were able to probe the lexical and sublexical language pathways separately. The real words that we used were exception words; these are words that are pronounced differently than standard phonological rules. These are best decoded by the neural networks that decode whole word, also known as the lexical pathway. Breaking down exception words in a letter-by-letter fashion often leads to the wrong rhyme decision. For example, the word “mint” does not rhyme with “pint” although phonological rules would predict that they rhyme. Conversely, non-words are novel letter strings that can be pronounced using standard phonological rules. Non-words are best processed in a letter-by-letter fashion since there is no way to recognize the entire word by sight (e.g., “leet”). These words need to be processed by the sublexical pathway, which is predicted to be distinct from the lexical pathway according to the dual-route model. To parallel these visual rhyme tasks, we also tested rhyming of analogous pairs of words or pairs of non-words presented auditorily.

Thus, both auditory processing pathways responsible for word and phonological processing and visual language networks responsible for orthographic and phonological decoding were investigated. To investigate differential organization of neural language pathways, functional images weighted by subject performance provide a representation of differential functional organization in neural pathways. This could provide significantly more information than simply demonstrating that language networks are different than typical in children with epilepsy (Vannest et al., 2013). Indeed, determining which portion of the language network is functional and dysfunctional is more helpful than simply showing that the network is abnormal. This is because the understanding of how neural networks are abnormally organized can be helpful in providing insight into the pathophysiological processes that result in cognitive dysfunction in children with epilepsy. Indeed, using both reading and listening skill, this study aims to better understand whether there is a differential response to simply hearing words rather as opposed to internally pronouncing the words. Such information could help in the future in the design of task for pre-surgical language mapping of the brain in children with epilepsy. Although some of the tasks that are used in this study are similar to MEG tasks used for pre-surgical language mapping (Papanicolaou et al., 1999, 2004; Merrifield et al., 2007), the set of task used in this study are complex and are not meant to replace pre-surgical language mapping. Indeed, non-invasive pre-surgical language mapping is very complicated and requires a careful comprehensive multimodal approach (Papanicolaou et al., 2014).

## Materials and Methods

### Participants

This study was conducted in accordance with the Declaration of Helsinki and the Institutional Review Boards at the authors’ affiliated institutions. A total of 15 children diagnosed with non-localized cryptogenic epilepsy were recruited from the neurology clinics. Most of the children were on antiepileptic mediations with most taking oxcarbazepine and fewer taking carbamazepine and valproic acid, and each one taking ethosuximide, lamotrigine, and intravenous immunoglobulin (IVIG).

All families were English speaking and all children received education in English speaking classrooms. Informed consent (and assent where appropriate) was obtained from the participants and their parents. The MEG recording session was conducted after informed consent. Participants had no contraindications for MEG or MRI and were compensated $20/h. Demographics are presented in Table 1.

**Table 1:** **Demographic, cognitive and language characteristics of the participants**.

Age	10 years 0 months (7.2 months)
Gender (male:female)	10:5 (67% male)
Full-scale non-verbal intellectual	91.7 (4.2)
Woodcock–Johnson III	
Word Attack	88.5 (5.9)
Letter–Word identification	84.5 (6.9)

*Values are represented as mean and standard errors except for gender, which is given in number of patients and % male*.

### Phonological awareness skill assessment

Lexical (whole-word recognition) and sublexical decoding (letter-by-letter, phonological decoding) was measured with Woodcock–Johnson III *Letter–Word Identification* and *Word Attack*, respectively (McGrew et al., 2007). Table 1 provides average performance for these skills while Table 2 outlines the specific score for each participant. All scores are standardized. Performance on reading-related skills varied in a continuous fashion from normal to subnormal values, thereby preventing linear relationships from arising simply due to large inter-subject differences. Performance on the two reading subscales were correlated as expected [*r*(13) = 0.89; *p* < 0.01]. For the most part, non-verbal intelligence varies from low normal to high normal with only one child within the mildly intellectually impaired range. Non-verbal intelligence demonstrated moderate correlations with the Letter–Word Identification [*r*(13) = 0.70; *p* < 0.01] and word attack [*r*(13) = 0.67; *p* < 0.01] reading subscales. Antiepileptic medications did not appear to be associated with performance.

**Table 2:** **Specific participant scores and medications**.

Gender	Age	Letter–Word Identification	Word Attack	Non-verbal intelligence	Antiepileptic medications
Female	7 years 4 months	124	118	111	None
Male	7 years 7 months	65	72	64	Carbamazepine
Male	8 years 3 months	74	68	85	Valproic acid
Female	8 years 4 months	64	67	80	Carbamazepine
Male	8 years 11 months	104	122	96	Oxcarbazepine
Male	8 years 11 months	107	102	113	Oxcarbazepine
Male	9 years 2 months	52	62	89	Valproic acid, IVIG
Female	9 years 8 months	93	97	87	None
Male	10 years 3 months	104	110	125	Oxcarbazepine
Female	10 years 6 months	88	87	85	Valproic acid
Male	11 years 3 months	77	76	93	Oxcarbazepine
Male	11 years 7 months	45	75	72	Lamictal
Male	12 years 5 months	110	102	105	Oxcarbazepine
Male	12 years 7 months	113	102	93	None
Female	14 years 2 months	99	95	79	Ethosuximide

### Stimulus presentation system

Auditory stimuli, stored as 8-bit monaural 22 kHz wav files, played through ER30 (Etymotics Research, Inc.) earphones. The frequency response of the system was flat within the normal speech range and the magnetic field artifact from the earphones was insignificant. The delay and jitter between the onset of the stimulus trigger and the auditory stimulus was 22 and 8 ms, respectively. All latency values were corrected for the delay.

### MEG language tasks

Participants performed two real word and two non-word tasks with one real word and non-word task being auditory and one real word and non-word task being visual during the MEG recording session. Each task was 6 min long with 3-min inter-task rest interval. Tasks were controlled by Presentation™(Neurobehavioral Systems, Albany, CA, USA) (Frye et al., 2009a, 2010). All participants were given the various tasks in the exact same manner, although the order of the presentation of each stimulus pair was randomized within each task. Thus, any effect of task order is the same across participants. Although counterbalancing the tasks was considered in the experimental design, without a large sample size, the authors felt that it would simply be a confounding factor.

Two 6-min auditory rhyme tasks were separated by 3 min as described in our previous study (Frye et al., 2010). The tasks required the participant to determine if two consecutively presented words rhymed. The real word task presented high frequency non-exception (42%) or exception (58%) real words. Non-exception words are orthographically decoded using regular phonological rules; examples include “tint,” “hint,” “lint,” and “mint.” Exception words require non-regular phonological rules that must be memorized. The exception word that corresponds to the preceding example is “pint.” The non-word task presented novel non-pseudohomophone pronounceable letter strings matched on similar length and unconstraint bigram frequency. Being novel non-pseudohomophone, these pronounceable novel non-words that did not sound like real words when pronounced. Specifically, they did not sound or look like any word in the Oxford Dictionary. Thus, these letter strings were designed so that they would not activate any semantic or lexical language pathways. The sound produced by the ER30 earphones was transmitted binaurally through two 5-m-long plastic hollow tubes to the ER13 Horn Foam eartips (Etymotics Research, Inc.) at an intensity of approximately 80 dB. The auditory word stimuli were produced by a native female English speaker with a flat intonation (duration between 321 and 848 ms; mean 535 ms). The words were digitized with a sampling rate of 22,000 Hz and 16-bit resolution. Sequential words were separated by a 500 ms interstimulus interval. The participant responded using a response pad. The randomly determined intertrial interval lasted from 2 to 3 s. A cross was displayed in the center of a screen and the participant was requested to fixate on the cross and to inhibit eye movements and blinking during the auditory stimulus presentation. Each task contained 68 trials with words rhyming in half of the trials.

As described in our previous study (Frye et al., 2009a), participants were required to determine if two consecutively visually presented words rhymed. Real words were either high frequency non-exception (42%) or exception (58%) words while non-words were non-pseudohomophone pronounceable letter strings. Most of the real word trials (81%) included at least one irregular word (e.g., tough) to be judged as rhyming with either another irregular word [e.g., “rough” (rhyme) vs. “though” (non-rhyme)] or a regular word [e.g., “fluff” (rhyme) or “flow” (non-rhyme)]. The remainder contained two regular words that were spelled differently (19%) [e.g., “plane” vs. “drain” (rhyme) or “flame” (non-rhyme)]. Hence, sounding out exception words phonetically will lead to errors. One must treat them as a well-known word, which is recognized as a “whole word” to know how to pronounce them. Non-word trials consisted of pronounceable letter strings that were not in the Oxford Dictionary. Pairs of non-words did not look alike, even when the pair rhymed [e.g., “leet” vs. “jeat” (rhyme) vs. “jyte” (non-rhyme)].

Real words and non-words had similar orthographic characteristics, including similar mean length and unconstrained bigram frequency. Each word was displayed in the center of the screen for 700 ms and the words were separated by a 500 ms interstimulus interval. The participant responded using a response pad. The randomly determined intertrial interval lasted from 2 to 3 s. A cross was displayed in the center of the screen when the words were not displayed. Each visual task contained 68 trials with words rhyming in half of the trials.

The visual real word task could not be accomplished by the memory of prior encounters with the word. The rhymes never had the same codas (e.g., both ending with “ate”). Therefore, the rhyme decision required reading the words to decide if they rhymed. The task could not be done by visual inspection. The visual non-word task could not be accomplished by past word experience. Instead, the participant had to phonologically decode (sound out) the letter string before a rhyme decision could be made. The auditory versions of the tasks were similar to the visual tasks, except that they were heard not seen.

### MEG data acquisition

Recordings were made in a magnetically shielded room with a WH3600 (4D Neuroimaging, San Diego, CA, USA) whole-head neuromagnetometer that consisted of 248 axial gradiometer superconducting quantum interference devices in a cryogenic dewar. The signal was continuously sampled at 500 Hz and filtered on-line with a bandpass filter between 0.1 and 150 Hz. Event-related fields (ERFs) were extracted and averaged after removing trials during which eye movement or blink occurred. To exclude blinking and other artifacts, epochs with gradiometer signals exceeding 3000 fT/cm were removed. Typically, one or two MEG channels were excluded for each participant due to artifacts.

Four head position indicator coils were attached to the scalp. These coils were used to determine the position of the head relative to the sensor array. The coils’ positions were measured using a low-intensity magnetic field generated by each coil at the start of each run. The location of the head position indicator coils, fiducial points, and approximately 50 points outlining the participants’ scalp were recorded using a 3-D digitizer (Hämäläinen et al., 1993) prior to the MEG recording to facilitate later MRI–MEG alignment.

### Magnetic resonance imaging

Two sets of structural MRI images were acquired for each participant using a 3-T Siemens Sonata scanner (Malvern, PA, USA) with a high-resolution 3-D T1-weighted magnetization-prepared 180° radio-frequency pulses and rapid gradient-echo (MP-RAGE) sequence optimized for gray–white matter contrast differentiation. The two sets of scans were registered and averaged. The cortical white matter was segmented and the border between gray and white matter was tessellated, providing a representation of the cortical surface with ~150,000 vertices per hemisphere (Fischl et al., 2001). The folded tessellated surface was then “inflated” in order to unfold cortical sulci, thereby providing a convenient format for visualizing cortical activation patterns (Fischl et al., 1999).

### MEG source current estimation

The cortical currents underlying the measured MEG signals were estimated using a distributed source model, the l2 minimum-norm estimate (Hamalainen and Ilmoniemi, 1994). The sources were assumed to be anatomically constrained to the cortical surface reconstructed from the MRI (Dale and Sereno, 1993). The cortical surface representation was decimated to approximately 3000 vertices per hemisphere; thus, neighboring sources were separated by about 5–10 mm. The forward model was produced by calculating the signal expected at each MEG sensor from a source of unit amplitude at each vertex using the boundary element method (Hamalainen and Sarvas, 1989). To reduce the sensitivity of the solution to small errors in the alignment between the MRI and MEG, the sources were not assumed to be strictly perpendicular to the cortical surface, but instead a small loose orientation parameter value of 0.1 was used (Lin et al., 2006a). Depth weighting was incorporated into the minimum-norm solution to reduce the bias of the solution toward superficial sources (Lin et al., 2006b). An estimate of cortical current at each source was then calculated every 1.6 ms. This method has been developed to examine distributed activation throughout the cortex.

Dynamic statistical parameter maps (dSPM) were calculated to produce functional maps of cortical activity (Dale et al., 2000). The dSPMs are produced by normalizing the current estimate at each source for noise sensitivity. The dSPM values calculated without an orientation constraint are *F*-distributed with 3 and *n* degrees of freedom (DOF) whereas sources calculated with a strict perpendicular orientation are *F*-distributed with 1 and *n* DOF (Dale et al., 2000). Since we used a partial orientation constraint (Lin et al., 2006a), the source values are expected to be *F*-distributed with numerator DOF between 1 and 2, with a conservative estimate being a DOF of 1. Since the square root of a 1 and *n* DOF *F*-distribution is a *t*-distribution with *n* DOF, we interpreted the dSPM values with a *t*-distribution with *n* DOF. We have previously used simulations to verify the appropriateness of these parameters (Frye, 2007).

### Cortical activation analysis

Two types of activation maps were used for analysis, average maps and differential maps.

Average dSPMs were produced by averaging across participants to provide a summary of cortical activity. The average activation was depicted on a representative cortical surface onto which the cortical surface for each participant was morphed (Fischl et al., 1999). Each participant’s cortical surface was transformed onto a spherical representation and registered with the representative brain by optimally aligning sulcal and gyral features, resulting in a linear mapping matrix between the two surfaces. For each participant, the cortical current estimate was mapped onto the representative cortical surface and the maps were averaged across participants. Since the exact vertices at which source activity is represented are not consistent across participants, an iterative procedure was used to spatially average activity from neighboring vertices. This procedure is linear and preserves amplitude information. There were four maps; one each for the auditory and visual rhyme task.

To derive differential maps, we weighted each individual cortical map by individual performance similar to our previous work (Frye et al., 2008). The measure of performance used to create the differential maps was the individual’s scores. Since the Woodcock–Johnson III Letter–Word Identification Test uses real words and probes lexical systems, this subscale was used for the auditory and visual *real word* rhyme tasks. Since the score on the Woodcock–Johnson III Word Attack Test is a measure of sublexical phonological decoding performance, it was used as the measure of reading performance for the *non-word* auditory and visual rhyme tasks.

The performance scores across all participants were rescaled to produce a weighting scheme modeled after orthogonal contrasts. Weights were designed so that overall performance weights would sum to zero so that if all participants demonstrated equal activation, the activation would be zero. The range of the performance values across participants was scaled to range between 1 and −1 such that participants with better performance were given positive values while participants with worse performance were given negative values.

Magnetoencephalography data provide a temporal aspect of data that can be difficult to visualize in still images. To help visualize the important aspects of the data, movies of cortical activation were reviewed and latencies were selected with represented peak changes in cortical activation. Three different cortical views were inspected (lateral, medial, and ventral) and latencies were selected where the peaks of activation occurred for each view of the cortex. These images were then captured to provide a sequence of still images for the figures in the results. For the weighted images, positive values were represented in red while negative values were represented in blue. Positive values (red) represented greater cortical activation by better performing individuals. Negative values (blue) represented greater cortical activation by worse performing individuals.

## Results

### Average cortical activity associated with the four rhyme tasks

#### Auditory non-word rhyme task (see Figure 1)

Overall, the average maps demonstrated bilateral activation of the primary and secondary auditory areas along with activation of the superior temporal sulcus and insular cortex. Of special interest was the finding that early activation (~130 ms) was minimal in the left hemisphere and relatively greater in the right hemisphere. Over time, this activity appeared to have increased in the left hemisphere and decreased in the right hemisphere.

**Figure 1:**
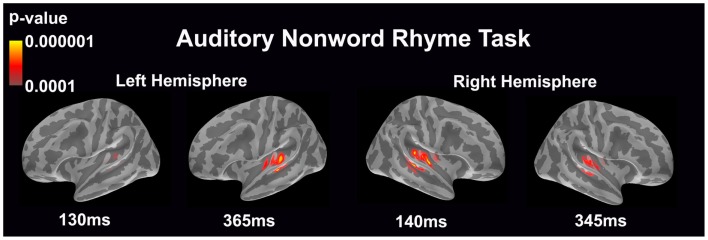
**Cortical activation during the auditory non-word rhyme task is shown**. Dynamic statistical parameter maps represent cortical activation averaged across 15 children with epilepsy with times representing latency from the onset of the auditory stimulus.

#### Auditory real word rhyme task (see Figure 2)

Similar to the auditory non-word rhyme task, the average maps demonstrated bilateral activation of the primary and secondary auditory areas along with activation of the superior temporal sulcus and insular cortex. Once again, earlier activation (~150 ms) was relatively lower in the left hemisphere as compared to the right hemisphere. Over time, this activity appeared to have increased in the left hemisphere and decreased in the right hemisphere.

**Figure 2:**
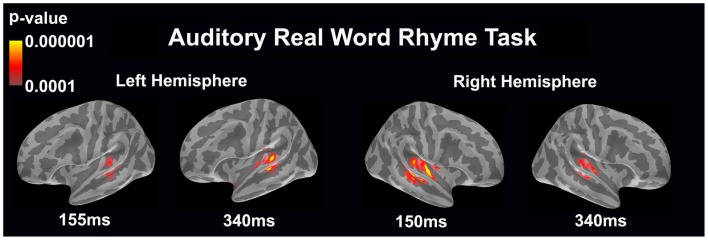
**Cortical activation during the auditory real word rhyme task is shown**. Dynamic statistical parameter maps represent cortical activation averaged across 15 children with epilepsy with times representing latency from the onset of the auditory stimulus.

#### Visual non-word rhyme task (see Figure 3)

In the left hemisphere, relatively early activation (~110 ms) occurred in the primary and secondary visual cortices and the angular gyrus. This was followed by activation in the ventral occipitotemporal area, planum temporale, and supramarginal gyrus with continued activation in the angular gyrus at 200 ms.

**Figure 3:**
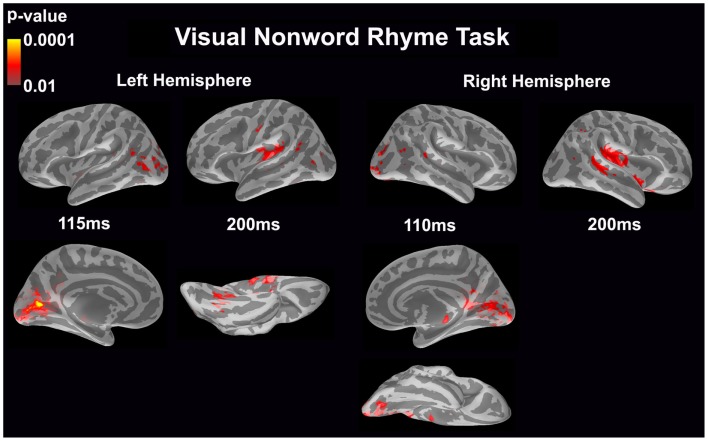
**Cortical activation during the visual non-word rhyme task is shown**. Dynamic statistical parameter maps represent cortical activation averaged across 15 children with epilepsy with times representing latency from the onset of the visual non-word stimulus, which was centered on the screen in front of the participant.

The right hemisphere pattern of activation paralleled the left hemisphere with relative early activation (~110 ms) of the primary and secondary visual cortices and angular gyrus. Activation then continued in the angular gyrus with additional activity in the superior temporal sulcus, planum temporale, and insular cortex at 200 ms.

#### Visual real word rhyme task (see Figure 4)

In the left hemisphere, relatively early activation (~110 ms) occurred in the primary and secondary visual cortices, the insular cortex, and the angular gyrus. This was followed by activation in the ventral occipitotemporal area, planum temporale, and supramarginal gyrus with continued activation in the angular gyrus at 200 ms. At 400 ms, activation then occurred in the supramarginal gyrus with continued activation of the insular cortex.

**Figure 4:**
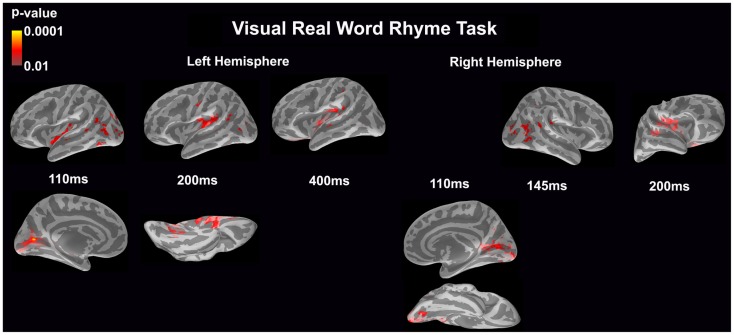
**Cortical activation during the visual real word rhyme task is shown**. Dynamic statistical parameter maps represent cortical activation averaged across 15 children with epilepsy with times representing latency from the onset of the visual real word stimulus, which was centered on the screen in front of the participant.

In the right hemisphere, relative early activation (~110 ms) of the primary and secondary visual cortices was followed by later activation of the angular gyrus and planum temporale at 145 ms. This was followed by activation of the superior temporal sulcus, planum temporale, and insular cortex at 200 ms.

#### Performance associated cortical activity

Differential maps are an excellent method for determining which patterns of cortical activation vary according to differences in accuracy of performance. This is important as key areas that might be important for accurate processing of language may not be revealed in average maps because of the great variation in activation due to differences in subject performance.

#### Auditory non-word rhyme task (see Figure 5)

Differential activation was related to both better and worse performance. In the left hemisphere, better performance was related to early (40 ms) engagement of secondary auditory areas such as the planum temporale as well as the superior temporal sulcus and later (385 ms) activation of the insular cortex, planum temporale, superior temporal sulcus, and areas in or near the supramarginal gyrus. In contrast, worse performance was associated with greater activation in the area of the planum temporale at 195 ms in the left hemisphere. In the right hemisphere, worse performance was associated with early (90 ms) and slightly later (190 ms) activation of the secondary auditory areas. In contrast, better performance was associated with greater activation in the superior temporal sulcus at 190 and 385 ms and the subcentral gyrus and insular cortex at 385 ms in the right hemisphere.

**Figure 5:**
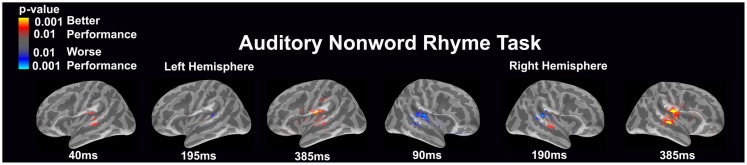
**Performance-dependent activation of neural pathways during the auditory non-word rhyme task is shown**. Red represents activation associated with better performance while blue represents activation associated with worse performance. Performance was based on the individual participant’s non-word reading ability as indexed by the scaled score on the Woodcock–Johnson III Word Attack subtest.

#### Auditory real word rhyme task (see Figure 6)

Differential activation was related to both better and worse performance. Interestingly, in the left hemisphere, worse performance was related to more activation of the planum temporale at both 185 and 385 ms, while better performance was related to more activation in the anterior superior insular cortex near the inferior frontal gyrus, the orbital frontal gyrus and sulcus, as well as the frontomarginal sulcus. For the right hemisphere, significantly worse performance was associated with early (60 ms) activation of the primary and secondary auditory areas including the planum temporale as well as slightly later (190 ms) activation in the planum temporale. Better performance was associated with greater later (365 ms) activation in insular area, particularly around the inferior frontal gyrus and the orbital frontal gyrus and sulcus.

**Figure 6:**
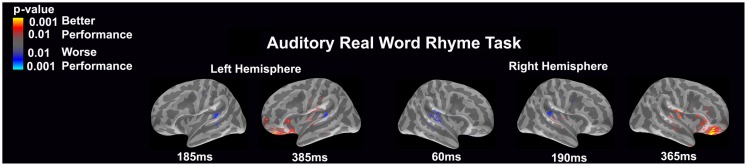
**Performance-dependent activation of neural pathways during the auditory real word rhyme task is shown**. Red represents activation associated with better performance while blue represents activation associated with worse performance. Performance was based on the individual participant’s real word reading ability as indexed by the scaled score on the Woodcock–Johnson III Letter–Word Identification subtest.

#### Visual non-word rhyme task (see Figure 7)

Differential activation was related only to better performance, but not worse performance. In the left hemisphere, better performance was associated with greater early activation (125 ms) in the anterior occipital sulcus and inferior occipital gyrus (seen on the lateral view of the cortex) and later activation (215 ms) of the occipitotemporal gyrus and lateral occipitotemporal sulcus (seen on the ventral cortical view) as well as the insular cortex and lateral temporal fissure (seen on the lateral view of the cortex). Following this activation, better performance was found to be associated with greater activation in the supramarginal gyrus, insular cortex, superior temporal sulcus, and planum temporale at 410 ms (seen on the lateral view of the cortex). The right hemisphere demonstrated similar activation to the left hemisphere, but this activation ended earlier. In the right hemisphere, better performance was associated with greater activation in the medial occipitotemporal sulcus (seen on the ventral cortical view) as well as the supramarginal gyrus, insular cortex, planum temporale, and superior temporal sulcus at 210 ms.

**Figure 7:**
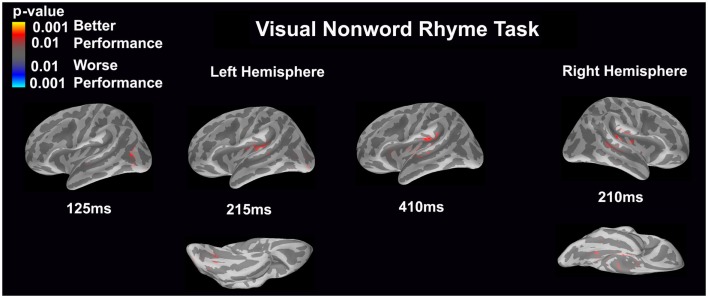
**Performance-dependent activation of neural pathways during the visual non-word rhyme task is shown**. Red represents activation associated with better performance while blue represents activation associated with worse performance. Performance was based on the individual participant’s non-word reading ability as indexed by the scaled score on the Woodcock–Johnson III Word Attack subtest.

#### Visual real word rhyme task (see Figure 8)

Differential activation was significantly related only to better performance, but not worse performance. In the left hemisphere, better performance was associated with greater early activation (135 ms) in the anterior occipital sulcus (seen on the lateral view of the cortex) and slightly later activation (195 ms) of the occipitotemporal gyrus (seen on the ventral cortical view). Following this early activation, better performance was found to be associated with greater activation in the supramarginal gyrus and insular at 260 and 430 ms (seen on the lateral view of the cortex) as well as superior temporal sulcus activation at 430 ms. The right hemisphere demonstrated similar activation to the left hemisphere, but this activation ended earlier. In the right hemisphere, better performance was associated with greater early activation (140 ms) in the anterior occipital sulcus (seen on the lateral view of the cortex) and slightly later activation (171 ms) of the medial occipitotemporal sulcus (seen on the ventral cortical view). This was also followed by greater activation in the supramarginal gyrus, insular cortex, planum temporale, and superior temporal sulcus at 200 ms.

**Figure 8:**
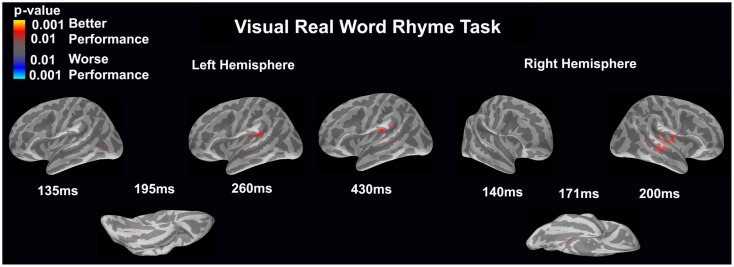
**Performance-dependent activation of neural pathways during the visual real word rhyme task is shown**. Red represents activation associated with better performance while blue represents activation associated with worse performance. Performance was based on the individual participant’s real word reading ability as indexed by the scaled score on the Woodcock–Johnson III Letter–Word Identification subtest.

## Discussion

Childhood epilepsy is associated with delays in development, including a high incidence of problems with language development (Benn et al., 2010). In this study, we used whole-head MEG to study patterns of organization of the neural pathway associated with abnormalities in language development in 15 children with non-localized cryptogenic epilepsy. Our sample had average full-scale non-verbal intelligence.

We had two goals. The first goal was to characterize patterns of recorded evoked fields activated during the performance of four kinds of rhyming tasks: real word and non-word stimuli presented in either the visual or auditory rhyme modality. We used whole-head MEG to examine the time course of activity in these children with cryptogenic epilepsy to see whether any of their activation patterns were anomalous as compared to those reported in the normal population. Results indicated that children with cryptogenic epilepsy, during the initial stages of processing the input, manifest a reversed pattern of hemispheric activation. At ~130 ms during the auditory non-word rhyme task and at about 150 ms in the auditory real word task, there was greater right than left hemispheric activation. During the remaining part of the recording, lateralization reversed back to the expected pattern of left greater than right hemispheric activation. No such reverse asymmetry was seen in either the visual non-word or real word rhyme task. In both the left and right hemispheres, early activation (~110 ms) occurred in the primary and secondary visual cortices and the angular gyrus, and angular gyrus activation continued bilaterally at 200 ms. What was noteworthy was that in the left, but not the right, hemisphere at 400 ms, activation persisted in both the supramarginal gyrus and the insular cortex.

The second goal was to use standardized measures [the Woodcock–Johnson III subtests of sight word reading (relevant to processing real words)] and word attack (relevant to phonological decoding on non-words). We then used performance information from these tests of language ability to weight dSPM. This analysis provided insight into function and dysfunction of language networks in childhood.

For both the real word and non-word auditory rhyme tasks, lower performance on language testing was related to greater engagement of the right primary and secondary auditory cortices, particularly early activation of these areas. This suggests that children with cryptogenic epilepsy may have experienced factors that led to reorganization of cerebral laterality.

For the non-word rhyme task, but not the real word rhyme task, better performance was associated with early engagement of the left planum temporale as well as the superior temporal sulcus, areas that are critical for decoding these detailed aspects of phonological processing of auditory language. Indeed, non-word auditory rhyme tasks require greater decoding of auditory syllables and greater reliance on the precise timing of frequency changes in speech than when a familiar word is decoded. These data suggest that, in children with epilepsy, incomplete lateralization of the low-level auditory language regions results in decreased ability to distinguish important aspects of language and interfere with language processing. These data further suggest that there is a temporal aspect to engagement of these areas such that early activation of these areas in the right hemisphere might interfere with accurate processing of speech. Further studies will need to determine how epileptic activity interferes with neural pathway development to result in these changes in neural organization.

Interestingly, relatively later cortical activation was related to better performance for both auditory tasks, but the areas involved in this relatively later activation were different for the two tasks. For the non-word rhyme task, greater relatively later activation in areas in and around the insular cortex, superior temporal sulcus, supramarginal gyrus, and subcentral gyrus in both hemispheres was related to better performance. While, for the real word rhyme task, better performance was associated with relatively later greater activation in and around the inferior frontal gyrus and the orbital frontal gyrus and sulcus. This may reflect the different processes involved in non-word vs. real word tasks as processing real words most likely require retrieval from long-term memory stores while non-words most likely require greater activation of phonological decoding networks.

For both visual rhyme tasks, better performance was related to greater general activation of specific brain areas. In particular, better performance was associated with early activation of the left anterior occipital sulcus followed by activation of the bilateral occipitotemporal gyrus. Around 200 ms for both rhyme tasks, better performance was associated with greater activation in the right supramarginal gyrus, insular cortex, planum temporale, and superior temporal sulcus and left peritemporal (supramarginal gyrus for the real word rhyme task and lateral temporal fissure for the non-word rhyme task) and insular. Relatively, later activation (~400 ms) was associated with better performance in the supramarginal gyrus, insular cortex, planum temporale, and superior temporal sulcus areas for the non-word rhyme task and the superior temporal sulcus for the real word rhyme task. Thus, both visual rhyme tasks showed that better performance was associated with relatively later left hemisphere activation, although the exact areas of this activation were slightly different for each task. This different late activation across the two tasks most likely represents the greater recruitment of basic phonological decoding and storage networks used to decode novel combinations of phonological information.

The pattern of brain activation associated with better performance in the visual rhyme tasks is consistent with neural networks associated with visual rhyme tasks seen in normal readers (Frye et al., 2009a, 2010; Rezaie et al., 2011a,b,c; Fisher et al., 2012; Simos et al., 2013). This may suggest that children with epilepsy with good visual word decoding skills have more typical reading networks. In contrast, during non-word auditory decoding, rapid changes in formant frequencies must be tracked, a process known to be superior in the left rather than right hemisphere. A history of cryptogenic epilepsy may be sufficient to disrupt early activation of the left hemisphere. The reason for the poor development of the neural networks for reading in some children with epilepsy is not clear but further studies, potentially longitudinal, may be helpful in understanding the factors, which interrupt the dynamic development of these networks in children with epilepsy. Some areas which might be important for the performance of such language tasks did not manifest differential activation in our study; these areas include the posterior cingulate (Binder et al., 2009), the hippocampus (Shtyrov, 2012) the medial temporal lobes (Binder et al., 2009), and the anterior cingulate gyrus (Bush et al., 2000). While differential activation was not found in these areas related to language, we did not examine them specifically, so our data do not support or refute the role of these areas in language or in the tasks used in this study. Interestingly, greater activation in the insula, an area that is inconsistently associated with language (Ibanez et al., 2010), was found to be positively related to performance. Further studies specifically examining these areas may be able to better understand their role in specific language tasks.

Previous studies have suggested that there is a central core to the neural network of the brain, which appears to be the hub for separate brain modules (Hagmann et al., 2008). Anatomically, these regions include the posterior cingulate cortex, precuneus, cuneus, paracentral lobule, cingulate isthmus, the banks of the superior temporal sulcus, and inferior and superior parietal cortex, in both hemispheres. For the most part, activations in these areas were not differentially related to performance in the participants examined in this study. The only exception is the bank for the superior temporal sulcus in which greater activation was associated with better performance. Thus, this appears to suggest that any disruption of the network central core of the brain does not appear to be associated with language disruption in children with cryptogenic epilepsy. Suggestion that disruption in the language network is more related to how the non-core modules temporally interact.

Overall, these data suggest that disruptions in lateralization of activation during particular spatiotemporal patterns of brain activation are associated with better and worse performance on language tasks in children with epilepsy. Using visual and auditory non-word and real word rhyme tasks, we demonstrate in this study that better performance on the language tasks were associated with particular patterns of lateralization consistent with previous studies in childhood epilepsy (Bedoin et al., 2011). However, this study further demonstrates that better performance is associated with specific spatiotemporal patterns of activity lateralization, including periods when left hemisphere activity extends beyond activation of the right hemispheres. For the auditory rhyme task, early activation of the right hemisphere was associated with worse language task performance. Thus, it is not only the relative amount of activity that is lateralized to the left or right hemispheres, but the relative timing of when such activity occurs. Interestingly, many of the cortical areas that are classically associated with language in the left hemisphere were also activated in the right hemisphere and when activated in the right hemisphere they were associated with better language performance as long as the temporal activation occurs within a specific temporal window.

This study did have some limitations that result in a limited applicability and generalizability of the findings. First, a control group was not used in this study, making the comparison to typically developing individuals indirect. Further studies with direct comparisons to typically developing individuals would be helpful. Second, many of the participants were taking antiepileptic medications. While there does not appear to be any correspondence between performance and antiepileptic medication, such medications can alter the dynamics and timing of neuronal activity, potentially leading to changes in the temporal dynamics of brain activation. Thus, the information of these treatments on our findings is not known. Third, we have used the same tasks for participants with a wide range of abilities. Given that task difficulty can confound the interpretation of MEG data (Simos et al., 2011; Fisher et al., 2012), the individual selection of tasks based on ability may be a more optimal design for future studies. Fourth, our method of weighting, while providing good information does not allow for detailed statistical analysis. Further studies using region of interest analysis and linear modeling may provide some additional quantitative information (Frye et al., 2008). Fifth, we have not considered the specific course of the epilepsy in these patients. In many cases, exact source localization is difficult to localize and requires intensive study using a multimodal approach. Even with intensive study, the source of epileptiform discharges is vague and the extent to which it results in neural disturbance can be widespread and include subcortical areas. Greater information about a localized source may have been helpful in the children examined but was not possible because of the nature of their epilepsy.

## Conclusion

This study demonstrates that children with non-localized cryptogenic epilepsy have some significant differences in the lateralization of their language neural networks that is related to deleterious performance on reading tasks. This suggests that any delays in language development may be related to disorganized or inefficient neural networks. The relationship between abnormalities in neural network organization and epilepsy is not clear. Presumably, factors associated with epilepsy, such as seizures, ongoing epileptic discharges, and antiepileptic drug treatment disrupt the ability of language networks to properly develop. Alternatively, it is possible that the same neuropathological processes that result in epilepsy also prevent the proper development of neural pathways as studies have found deficits in children with epilepsy at diagnosis (Jackson et al., 2013). Further, potentially longitudinal, studies will be needed to be conducted in order to better understand the etiology of developmental language delays in children with epilepsy. Clearly, these data emphasize some of the difficult and complicated issues that contribute to language localization. These data have implications for pre-surgical language mapping and suggests that the use of a wide range of language tasks during language mapping studies may provide a broader picture of the networks involved in language in children with epilepsy.

## Conflict of Interest Statement

The authors declare that the research was conducted in the absence of any commercial or financial relationships that could be construed as a potential conflict of interest.
